# Prevalence of skin disorders among psychiatric inpatients

**DOI:** 10.1192/j.eurpsy.2024.1008

**Published:** 2024-08-27

**Authors:** M. Turki, W. Abid, O. Khardani, A. Mellouli, M. A. Megdiche, N. Halouani, S. Ellouze, J. Aloulou

**Affiliations:** psychiatry B department, Hedi Chaker university hospital, sfax, Tunisia

## Abstract

**Introduction:**

The interface between dermatology and psychiatry is complex and of clinical importance. Skin disorders in psychiatric inpatients are common, serious and under diagnosed.

**Objectives:**

The aim of our study was to assess the prevalence and profile of several skin diseases observed in psychiatric inpatients.

**Methods:**

We conducted a cross-sectional study in the period from october,13 2023 to october,20 2023, among psychiatric male inpatients, hospitalized in psychiatry B department of the Hedi Chaker University Hospital (Sfax, Tunisia). We collected sociodemographic and clinical data using a pre-established form.

**Results:**

Over a period of a week, 35 patients were included in our study. The mean age of patients was 39,97 years. Among them, 80% were single and 14,3% were married. Addictive behaviors were reported in 74,3% of cases. The level of hygiene was good in 74,3% of patients. The three most common psychiatric diagnoses were schizophrenia (31,4%), followed by bipolar disorder (28,6%) and schizoaffective disorder (25,7%). We recorded 13 cases of skin diseases (37,2% of patients). Dermatological lesions were dominated by traumatic origin in 14,3% of cases. They were of infectious origin in 11,4% of cases, immunoallergic in 8,6% and parasitic in 2.9%.

**Conclusions:**

The prevalence of skin diseases is high in psychiatric inpatients, for whom proper skin care is necessary to improve their quality of life.

**Disclosure of Interest:**

None Declared

